# Effect of Zn-Rich Wheat Bran With Different Particle Sizes on the Quality of Steamed Bread

**DOI:** 10.3389/fnut.2021.761708

**Published:** 2021-12-10

**Authors:** Huinan Wang, Anfei Li, Lingrang Kong, Xiaocun Zhang

**Affiliations:** ^1^Agronomy College, State Key Laboratory of Crop Biology, Shandong Agricultural University, Taian, China; ^2^Key Laboratory of Food Nutrition and Safety, Ministry of Education, College of Food Science and Engineering, Tianjin University of Science and Technology, Tianjin, China

**Keywords:** Zn biofortification, Zn content, wheat bran, wheat flour, viscoelasticity, steamed bread

## Abstract

Bran is the main by-product of wheat milling and the part of the grain with the highest Zn content. We investigated the effects of the particle sizes (coarse, D50 = 375.4 ± 12.3 μm; medium, D50 = 122.3 ± 7.1 μm; and fine, D50 = 60.5 ± 4.2 μm) and addition level (5–20%) of Zn-biofortified bran on the quality of flour and Chinese steamed bread. It was studied to determine if the Zn content of steamed bread could be enhanced without deleterious effects on quality. Dough pasting properties, such as peak viscosity, trough viscosity, final viscosity, breakdown, and setback, decreased significantly as the bran addition level was increased from 5 to 20% but did not significantly differ as a result of different bran particle sizes. Bran incorporation significantly increased hardness, gumminess, chewiness, and adhesiveness, whereas the springiness, cohesiveness, and specific volume of steamed bread decreased with the increase in bran addition. The optimal sensory score of steamed bread samples in the control and Zn fertilizer groups were obtained under 5% bran addition resulting in comparable flavor, and texture relative to control. Meanwhile, the Zn content of the steamed bread in the Zn fertilizer group was 40.2 mg/kg, which was 55.8% higher than that in the control group. Results indicated that adding the appropriate particle size and amount of bran would be an effective and practical way to solve the problem of the insufficient Zn content of steamed bread.

## Introduction

Zn is one of the most essential trace elements that are closely related to human health. It is a component of more than 100 enzymes in the human body and a participant in the synthesis of nucleic acids and protein. Zn deficiency seriously affects the secretion of enzymes and hormones, thus endangering human health. More than 50% of the world's agricultural soils are deficient in available Zn (1). Approximately 30% of the global population is affected by insufficient Zn intake (2). Every year, ~20% of deaths in children, especially children in developing countries, under the age of 5 years die from various diseases caused by Zn, Fe, and/or I deficiency (3). The WHO regards Zn deficiency as a major risk factor and an invisible killer threatening human health.

Wheat is one of the major food crops that is considered to be an important candidate for Zn biofortification. In China, the Zn content of wheat grain grown in major wheat-producing areas is <30 mg/kg (4), which is lower than the international recommended standard of 40–60 mg/kg (5). Thus, this level cannot satisfy the Zn demand of the population that consumes wheat as their staple food. Moreover, soils in the main wheat-producing areas in China are potentially deficient in Zn; this condition seriously affects the accumulation of Zn in wheat. The foliar spraying of Zn fertilizers is an effective measure for increasing the Zn content of wheat grains rapidly (6).

The processing of wheat for consumption always includes two main steps: grinding wheat grain into flour and food preparation. During these processes, bran, which has the highest Zn content, is often discarded, resulting in the large loss of Zn from wheat grains. Therefore, bran can be applied in the processing of steamed bread to improve the content of Zn and increase the utilization rate of Zn in wheat grains. Wheat grain is composed of three components: the bran, embryo, and endosperm. The endosperm is the main ingredient of flour, and the bran is the main by-product of flour processing. Studies have shown that the content of metal elements, such as Zn, in wheat grain embryos, aleurone layers, and seed coats is drastically higher than that in the endosperm (7). The Zn content of the embryo and aleurone layer is approximately 150 mg/kg, whereas that of the endosperm is only 15 mg/kg (8). These values show that wheat bran has a considerably higher Zn content than flour and is rich in dietary fibers, good quality proteins, and antioxidants. Therefore, the reasonable application of bran in steamed bread processing can not only improve the Zn content of steamed bread and the utilization ratio of bran, it is also a safe and effective means of daily Zn supplementation. However, the addition of wheat bran often affects the structure and sensory quality of the resulting food and reduces consumer acceptance. Some researchers have shown that bran particle size has a significant effect on dough properties and product quality, such as the use of MWB (microparticulated wheat bran) could improve the texture as well as the specific volume of whole wheat bread (9). The effects of bran addition levels on noodles (10, 11), baked and steamed bread (12–14), and biscuits have been studied (15, 16). Zhang and Li (11) reported that addition of fine bran (0.21 mm) at 5–10% or medium bran (0.53 mm) at 5% in wheat flour, it is possible to satisfactorily produce fiber-rich dry white Chinese noodle. Sozer et al. (16) observed that particle size reduction of bran increased the biscuit hardness and decreased the starch hydrolysis index of biscuits. Steamed bread is the traditional staple food in China (17). It accounts for approximately 40% of national total wheat consumption (18). Steamed bread plays an important role in the diet structure of China. However, the fortification of steamed bread with Zn-rich wheat bran has not been systematically studied. The current study focused on the effect of adding bran with different particle sizes and proportions on the quality of wheat flour and dough properties, including Zn content and pasting, rheological, and structural properties. We anticipated that the results of this study would provide a new scheme for the rational use of wheat bran and scientific information enabling the quality improvement of Zn-fortified products.

## Materials and Methods

### Plant Materials and Growth Conditions

The winter wheat cultivar used for the study was “Shannong 29,” which was bred by Shandong Agricultural University. The field experiment was conducted at the Shandong Agricultural University Research Farm (117°16′69.52′′N, 36°16′67.09′′E). This area has a warm and semihumid continental monsoon climate, an annual average rainfall of 680 mm, and an annual mean temperature of 12.8°C. During the growing season, Zn fertilizer was sprayed at the jointing, flowering, pre-grouting, and late filling stages for the Zn biofortification of wheat. The foliar treatments were as follows: (1) foliar spraying of water as a control and (2) spraying of ZnSO_4_·7H_2_O (0.4%, w/v) containing 0.01% (v/v) Tween 20 as a surfactant. All the fertilization treatments were performed after sunset. Each plant plot had an area of 50 m^2^ (5 m × 10 m) with three replications. The line spacing was 0.25 m, and the plant spacing was 0.02 m.

### Preparation of Wheat Flour Samples

After harvesting, part of the samples was milled into flour by using a MM400 Hybrid Ball Mill (Retsch, Beijing, China) for the determination of the Zn concentration of whole grains. The other wheat samples were milled into three core powders (1M, 2M, and 3M), three hide powders (1B, 2B, and 3B), and bran by using a MLU-202 Automatic Laboratory Mill (Buhler, Inc., Wuxi, China) in accordance with the AACC-approved method 26-21A (19).

### Preparation of Bran Samples With Different Particle Sizes

The bran collected from the mill was sifted into coarse bran (particle size of 2–2.5 mm) by using a JJSD sieve shaker (Shanghai Jiading Ltd. equipped different sized screens) with differently sized screens. Whole wheat bran was ground by using a fine grinder (KC-701, Kaichuang Instruments Co., Ltd., Beijing, China) into fine-ground bran. The bran samples were then divided into three equal portions. Two were subsequently ground into medium and fine particles by using a Perten 3100 laboratory mill (PerkinElmer Instruments Co., Ltd.) equipped with meshes of different sizes. Coarse bran (D50 = 375.4 ± 12.3 μm) was superfinely ground into medium (D50 = 122.3 ± 7.1 μm) and fine (D50 = 60.5 ± 4.2 μm) bran. The bran samples with different particle sizes were added to the flour at 0, 5, 10, 15, and 20% levels (on a 14% moisture basis). In this work, whole flour was the flour that contained whole grain. Standard flour was a blend of the 1M, 2M, 3M, 1B, 2B, and 3B fractions and was similar to commercially available flour. Coarse flour was a mixture of standard flour and coarse bran. Medium flour was a mixture of standard flour and medium bran. Fine flour was a mixture of standard flour and fine bran.

### Nutrient Analysis

The samples were digested with HNO_3_-H_2_O_2_ (4, 2 ml) in a microwave digester reaction system (Multiwave3000, Brabender, Germany). Then, the Zn content of the digested solution was determined through inductively coupled plasma optical emission spectroscopy (X Series 2, Thermo Fisher, USA). After use, all the instruments used in the experiment were soaked in 20% nitric acid overnight, then rinsed with deionized water and dried. The moisture content, ash content, and falling number of the flour samples were determined in accordance with the Chinese National Standards GB/T 5497-1985, GB/T 5009.4-2003, and GB/T 10361-89, respectively. Protein content on a dry weight basis was determined through the Kjeldahl method in accordance with the International Approved Method 46-12 (19). The sedimentation value of dough was determined in accordance with the AACC International Approved Method 76-31 (19).

### Determination of Pasting Properties

The pasting properties of the samples were determined by applying a Rapid Visco Analyzer (RVA-Super 3, Newport Scientific, Australia) in accordance with the AACC International Approved Method 76-21 (19). The test was performed by using 3.5 g (14% moisture basis) blends from each sample mixed with 25 ± 0.1 ml of distilled water (corrected for compensation on a 14% moisture basis).

### Whiteness

A Minolta Chroma Meter (Model CR-400, Minolta Co., Osaka, Japan) was used to determine the whiteness of wheat flour and obtain L^*^, a^*^, and b^*^ values. L^*^ represents brightness and is a measure of black to white (0–100). Large L^*^ values are indicative of high whiteness and brightness. a^*^ stands for the red–green phase. A positive a^*^ value indicates redness, whereas a negative a^*^ value reflects greenness. b^*^ stands for the yellow–blue phase. If b^*^ is positive, then the sample is yellow. If b^*^ is negative, then the sample is blue.

### Gluten Content and Index

The gluten content and index were determined by using a Glutomatic 2200 system (M/S perter, Germany) in accordance with the AACC International Approved Method 38-12.02 (19). A total of 10 g of each dough sample was weighed out, and wet gluten was converted into 14% wet base.

### Preparation of Steamed Bread

Steamed bread was prepared with 200 g of flour or flour blended with bran, 2 g of yeast, and water (85% of Mixolab water absorption). Before dough mixing, the yeast was dissolved in water (35°C). Then, the yeast solution and flour were poured into a mixing machine (HL-110, Shaoguan Co. Ltd., Guangzhou, China) and mixed for 5 min. The mixed dough was sheeted 15 times on a tablet pressure machine to remove air bubbles and split into 100 g portions. Each chunk was rolled with heights of approximately 6 cm by hand and then fermented (35°C, 80% relative humidity) for 45 min in a cabinet. After fermentation, the buns were steamed for 20 min in an electric steamer and cooled for 5 min after turning off the fire.

### Sensory Evaluation

The steamed bread samples were cooled to room temperature for approximately 1 h before the measurement. The volume of steamed bread was determined by using the rapeseed displacement method. The specific volume was calculated from the volume-to-weight ratio of the steamed bread. GB/T21118-2007 with a few modifications was adopted for the sensory quality analysis of steamed bread. Specific volume, color, surface structure, appearance, internal structure, elasticity, toughness, stickiness, and scent were scored by six trained evaluators. The highest scores for each item were 20, 10, 10, 10, 15, 10, 10, 10, and 5, which provided a total score of 100 (the specific score was accurate to 0.1). The eating quality of the cooked steamed bread was subjectively evaluated by 16 (the male to female ratio was 1:1; the average age range is between 25 and 55.) trained panelists according to Chinese Standard Method GB/T 35991-2018.

### Textural Analysis

After sensory evaluation, the steamed bread was subjected to textural profile analysis (TPA) by using a TA XTPlus Texture Analyze apparatus (TA XT Plus; Stable Micro Systems Ltd., Godalming, Surrey, UK) equipped with a P/35 R probe. Before the test, the steamed bread was cooled to room temperature and then cut into 10 mm-thick slices. The two center slices were taken for TPA. The test parameters were as follows: pretest speed of 1.0 mm/s, test speed of 1.0 mm/s, post-test speed of 1.0 mm/s, compression ratio of 50%, trigger force of 5 g, interval time of 5 s, and data collection rate of 200 pps.

### Statistical Analysis

All data were collected at least in triplicate. The statistical analysis of the results was carried out with SPSS software (SPSS 19.0, SPSS Inc., Chicago, U.S.A.), and Duncan's test (*p* < 0.05) was used to compare significant differences among the samples.

## Results and Discussion

### Effect of Spraying Zn Fertilizer on the Zn Content of Wheat and Flour

**Figure 1:**
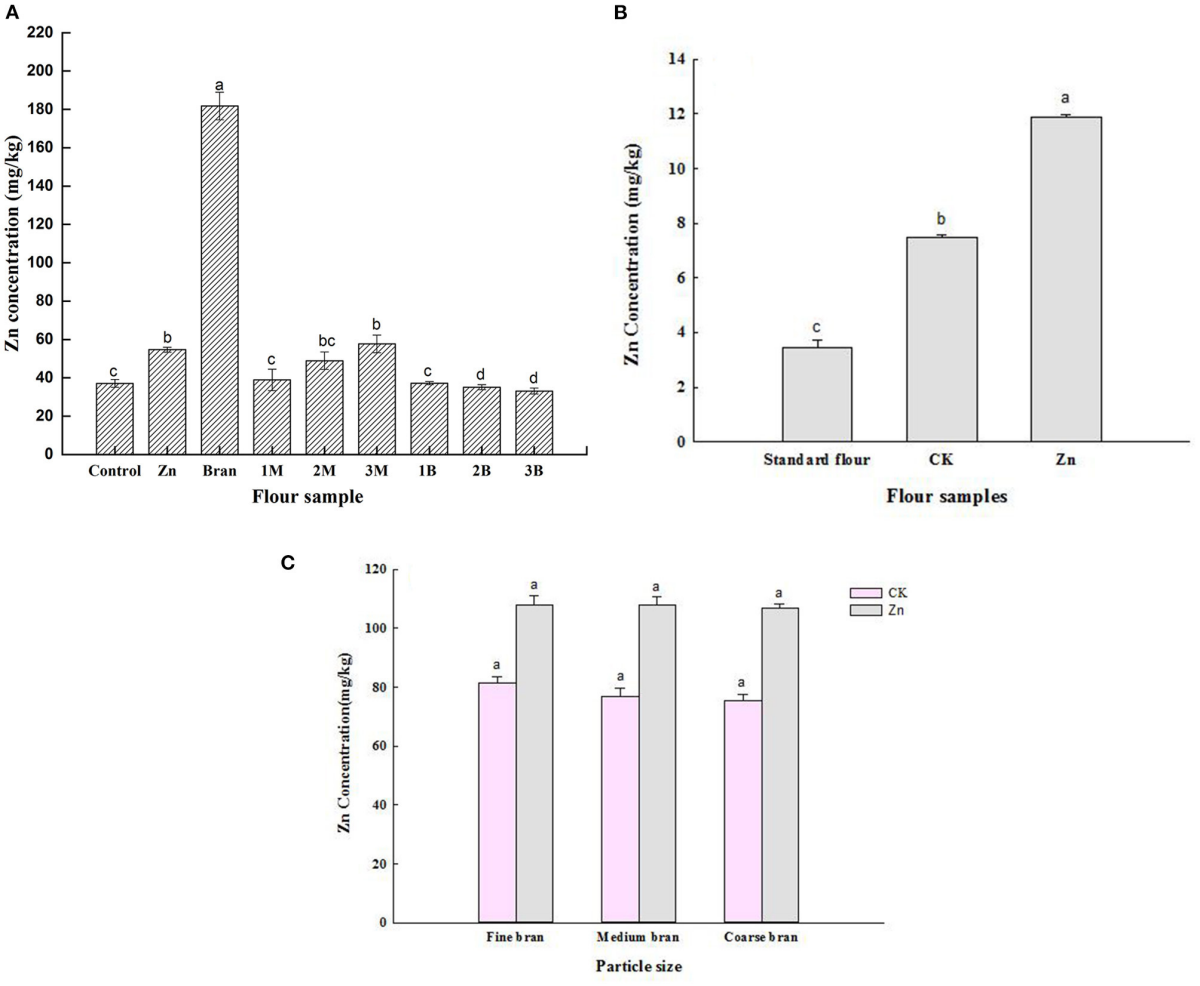
**(A)** Zn content of whole grain and different grain components. 1M: Core powder 1, 2M: Core powder 2, 3M: Core powder 3, 1B: Hide powder 1, 2B: Hide powder 2, 3B: Hide powder 3. Means with different letters (a–c) for each column showed significant difference (*P* < 0.05). **(B)** Zn content of different flour. Means with different letters (a–d) for each column showed significant difference (*P* < 0.05). **(C)** Zn content of bran with different particles.

The effects of spraying Zn fertilizer on the Zn content of whole grain and different grain components are summarized in Figure 1A. The average Zn content of the wheat grain in the control group was 37.1 mg/kg and that of the wheat grain in the Zn fertilizer group was 54.7 mg/kg. Compared with that of the control group, the Zn content of the wheat grain in the Zn fertilizer group had increased by 47.4%. Considering that bran had the highest Zn concentration, it was the part of the wheat grain wherein Zn mainly accumulated. Significant differences were found among the Zn contents of the three core powder flours (*p* < 0.05), which followed the order of 3M > 2M > 1M. By contrast, no significant differences were found among the Zn concentrations of the three hide powder flours. As can be seen from Figure 1B, the Zn contents of flour in the control group (7.5 mg/kg) and the fertilizer group (11.9 mg/kg) were significantly higher than those of the flour sold in markets (3.4 mg/kg) (*p* < 0.05). Meanwhile, the Zn content of the Zn fertilizer flour had increased by 58.7% compared with that of the control flour. These results showed that the foliar spraying of Zn fertilizer is an effective way for bioaccumulation (20). Figure 1C shows that the Zn concentration of bran with different particle sizes did not significantly differ (*p* > 0.05) between the CK and Zn groups, indicating that the processing of bran into different particle sizes would not affect Zn content. These results showed that Zn fertilization effectively increased the Zn concentrations of whole wheat grain relative to the control treatment, and bran was the most Zn-rich part of wheat grains.

### Effect of Bran Addition on Flour Quality

#### Effects of Bran Addition on the Physicochemical Properties of Flour

As can be seen from Table 1-1, after the addition of bran, the moisture content of the flour samples in the CK and Zn fertilizer groups gradually decreased with the reduction in bran grain size and significantly decreased with the increase in the addition amount of bran (*p* < 0.05). These results could be attributed to the following: The grinding of small grains required more time and consumed more energy than that of large grains. The heat generated by the processing equipment in this process may be an important reason for water loss. In addition, the whiteness of flour decreased significantly with the increase in bran addition (*p* < 0.05). Whiteness is the major sensory indicator, which is actively correlated with consumers' acceptance. Bran was brownish yellow, and the flour was milky white as usual. The color of the blends gradually deepened and darkened as the amount of bran added to the flour was increased. As can be seen from Table 1-2, with the increase in the addition level of the three kinds of bran, the a^*^ and b^*^ values increased and the L^*^ value decreased obviously. Dose-dependent lower L^*^ value indicated bran incorporation darkened the appearance of flour (21). Under the same additive amount, L^*^ decreased with the reduction in bran particle size in the control and Zn fertilizer blends because the even mixing of the small bran particles with the flour reduced the amount of light reflected by the flour surface and decreased brightness.

**Table 1-1:** The change of physicochemical index of flour after adding different bran.

**Bran granularity**	**Bran addition (%)**	**Moisture content (%)**		**Whiteness**	
		**CK**	**Zn**	**CK**	**Zn**
	0	13.94 ± 0.04a	13.87 ± 0.05a	73.85 ± 0.21a	73.40a
	5	13.87 ± 0.04ab	13.81 ± 0.01e	68.00b	68.9 ± 0.42b
Coarse bran	10	13.0.84 ± 0.06b	13.78 ± 0.05d	63.7 ± 0.14c	65.55 ± 0.35c
	15	13.82 ± 0.1bc	13.74 ± 0.06c	61.35 ± 0.35d	62.9 ± 0.42d
	20	13.75 ± 0.02c	13.71 ± 0.07b	58.65 ± 0.21e	60.95 ± 0.07e
	0	13.94 ± 0.04a	13.87 ± 0.05a	73.85 ± 0.21a	73.40a
	5	13.64 ± 0.01e	13.87 ± 0.06d	66.80b	68.6 ± 0.14b
Medium bran	10	13.47 ± 0.01d	13.56 ± 0.04cd	63.65 ± 0.07c	65.85 ± 0.07c
	15	13.19 ± 0.06c	13.11 ± 0.04b	60.90d	64.05 ± 0.07d
	20	13.08 ± 0.01b	12.49 ± 0.01c	58.2 ± 0.14e	62.80e
	0	13.94 ± 0.04a	13.87 ± 0.05b	73.85 ± 0.21a	73.40a
	5	13.31 ± 0.06d	13.33 ± 0.48a	68.55 ± 0.21b	69.00b
Fine bran	10	12.96 ± 0.14c	12.58 ± 0.02a	65.35 ± 0.21c	65.75 ± 0.07c
	15	12.73 ± 0.09b	12.59 ± 0.51a	63.35 ± 0.07d	63.60 ± 0.07d
	20	12.66 ± 0.01a	12.12 ± 0.04a	61.15 ± 0.21e	62.10e

*The same alphabets in right side of the same list show no significance (P > 0.05), on the contrary, having significance (P < 0.05)*.

**Table 1-2:** The color of the bran flour.

**Bran granularity**	**Bran addition (%)**	L*		a*		b*	
		**CK**	**Zn**	**CK**	**Zn**	**CK**	**Zn**
	0	90.53a	90.64a	−0.04a	0.09a	9.35a	9.26a
	5	88.56b	89.08b	0.65b	0.59b	10.49b	9.87b
Coarse bran	10	87.30c	87.92c	0.91c	0.91c	11.23c	10.52c
	15	86.23d	87.15d	1.32d	1.09d	11.64d	11.11d
	20	85.83e	86.46e	1.45e	1.28e	11.91e	11.32e
	0	90.53a	90.53a	−0.04a	−0.04a	9.35a	9.35a
	5	88.47b	89.01b	0.66b	0.59b	10.02b	9.56b
Medium bran	10	86.82c	87.92c	1.26c	0.91c	10.33c	10.03c
	15	85.48d	86.91d	1.59d	1.14d	11.2d	10.51d
	20	84.25e	86.05e	1.91e	1.28e	11.55e	10.96e
	0	90.53a	90.64a	−0.04a	0.09a	9.35a	9.26a
	5	88.83b	88.86b	0.44b	0.54b	9.62b	9.31b
Fine bran	10	86.79c	87.08c	1.06c	1.12c	10.08c	9.62c
	15	84.82d	85.31d	1.37d	1.63d	10.91d	10.43d
	20	83.99e	84.66e	2.02e	1.83e	11.37e	10.05e

*The same alphabets in right side of the same list show no significance (P > 0.05), on the contrary, having significance (P < 0.05)*.

**Figure 2:**
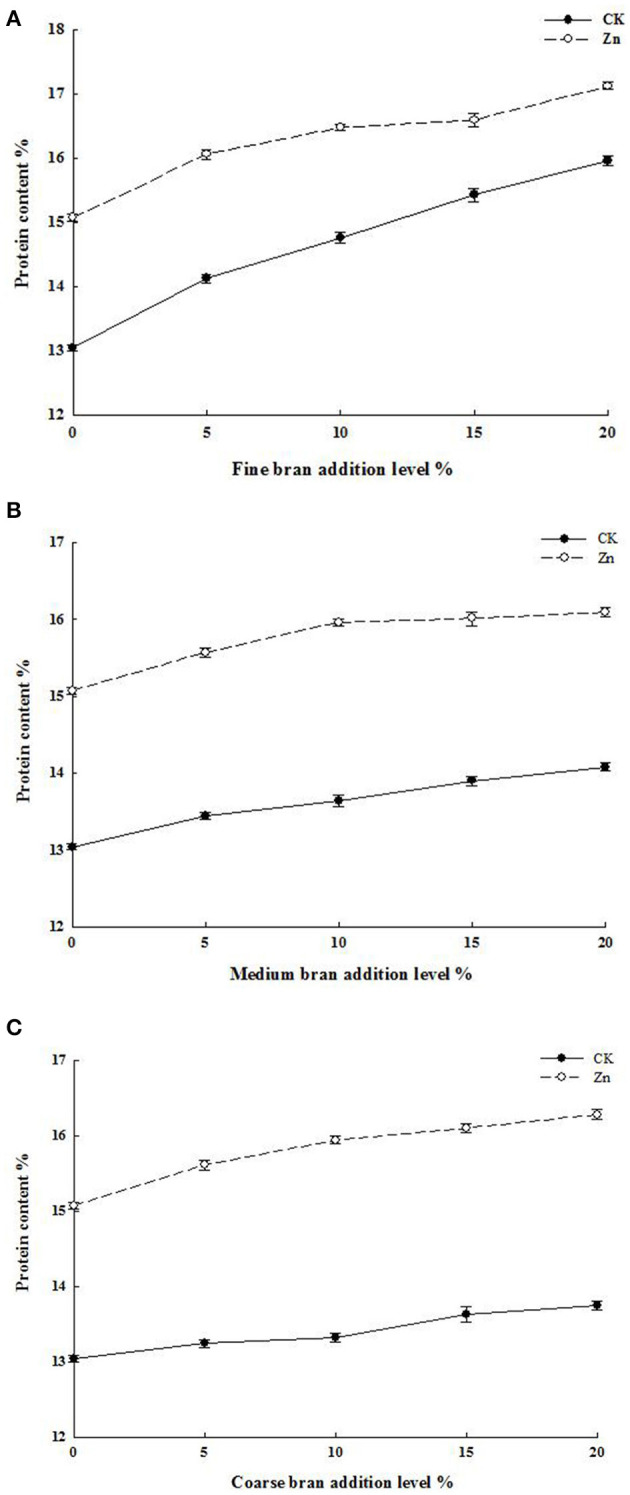
Protein content of flour containing different addition levels and particle sizes of wheat bran. **(A)** Fine bran; **(B)** Medium bran; **(C)** Fine bran.

The protein content of the samples is shown in Figure 2. With the addition of bran, the protein content of the CK and Zn fertilizer groups increased significantly (*p* < 0.05) and was positively correlated with bran addition level. Generally, wheat bran contains more than 15% high-quality proteins (22). It has been reported that there is a significant positive correlation between grain protein and zinc or iron concentrations (23, 24). In addition, the bran with large particle size retains more aleurone layers, while the aleurone layer contains high protein content. Therefore, the larger the grain size of the bran, the higher the protein content of the flour under the same amount of addition. Moreover, given that the high-quality protein content of bran exceeded 15% (22), the crude protein content of the flour increased after bran addition. Under the same addition level, the protein content of the mixed flour after the addition of coarse bran, medium bran, and fine bran reached 16.1, 16.3, and 17.1%, respectively.

#### Effects of Bran Addition on the Wet Gluten Content and Gluten Index of Flour

Gluten is not only a nutritional quality character but also a processing quality character. The quality of wheat depends on the quality and quantity of gluten. Therefore, wet gluten content and gluten index are important for evaluating the processing quality of wheat. Jian Zhang (25) showed that the wet gluten content and total protein content of flour are significantly and positively correlated. As can be seen from Table 2-1, 2-2, with the increase in the addition levels of coarse bran, medium bran, and fine bran, the content of wet gluten first increased and then decreased. The maximum wet gluten content of 38.57% was obtained when medium bran was added at the 5% level into Zn fertilizer flour. At the same particle size, the gluten index decreased significantly with the increase in the added amount of wheat bran (*p* < 0.05), indicating that the addition of bran could affect the formation of gluten in the dough. The possible reasons are as follows: on the one hand, although the protein content increased due to the presence of more bran, the bran protein was mainly dominated by globulin and albumin, and the gluten quality was poorer than that of endosperm protein; on the other hand, the presence of bran had an adverse effect on the gluten network, making it difficult for the gluten to form a strong network structure (26). The highest gluten index was obtained when the bran addition level was 5%, representing the best gluten quality. Moreover, the wet gluten content and gluten index of the flour in the Zn fertilizer group flour were higher than those of the flour in the control group. These results indicated that the application of Zn improved the gluten content and quality of flour.

**Table 2-1:** The sedimentation value of the CK bran flour.

**Flour**	**Bran granularity**	**Bran addition (%)**				
		**0**	**5**	**10**	**15**	**20**
	coarse	27.50 ± 0.71a	23.00b	13.00c	9.50 ± 0.71b	9.37 ± 0.71d
CK	medium	27.50 ± 0.71a	18.00b	16.50 ± 0.71b	14.50 ± 0.71b	12.10 ± 0.71c
	fine	27.50 ± 0.71a	17.50 ± 0.71b	14.50 ± 2.12b	15.50 ± 0.71b	14.00 ± 1.41b

*Those with the same letters in the same row indicated that the difference in the same period did not reach the significant level (P > 0.05), while those with different letters indicated that the difference reached the significant level*.

**Table 2-2:** The sedimentation value of the Zn bran flour.

**Flour**	**Bran granularity**	**Bran addition (%)**				
		**0**	**5**	**10**	**15**	**20**
	coarse	29.00a	24.5 ± 0.71b	19.00c	15.00d	10.50 ± 0.71e
Zn	medium	29.00a	19.5 ± 0.71b	12.00 ± 1.4c	16.00 ± 1.41d	12.00 ± 1.41d
	fine	29.00a	18.00b	11.00c	15.50 ± 0.71c	14.00 ± 1.41d

*Those with the same letters in the same row indicated that the difference in the same period did not reach the significant level (P > 0.05), while those with different letters indicated that the difference reached the significant level*.

#### Effects of Bran Addition on the Sedimentation Value of Flour

Sedimentation value is also an important index for evaluating flour quality. This index reflects the quantity and quality of gluten protein. A high sedimentation volume indicates strong gluten and vice versa. The sedimentation value is significantly related to the cooking quality of flour and depends on the hydration rate and hydration capacity of gluten protein. Upon mixing with water, the hydrophobic bonds, such as hydrogen bonds, of the gluten protein are broken, and the gluten molecule's hydration capability is enhanced such that the swollen flour particles form flocculent precipitates. As can be seen from Table 3 and consistent with the protein content of the bran flour in the two groups, the sedimentation value of the Zn fertilizer group was higher than that of the control. However, with the increase in the added amount of bran, the sedimentation values of the control and Zn groups decreased significantly (*p* < 0.05) likely because the addition of bran destroyed the network structure formed by gluten and reduced the quality of the dough. This result was consistent with the changes of gluten content and gluten index. The adverse effect of bran addition on gluten network formation could be explained by its role as a filler suspended in the dough (27).

**Table 3:** The gluten content in different bran flour.

**Bran granularity**	**Bran addition (%)**	**Wet gluten content (%)**		**Gluten index**	
		**CK**	**Zn**	**CK**	**Zn**
	0	35.64 ± 0.57b	37.65 ± 0.56b	72.85 ± 0.21a	73.40a
	5	37.64 ± 0.41ab	37.74 ± 0.64ab	68.65 ± 0.07b	68.70b
Coarse	10	38.31 ± 022a	38.45 ± 0.24a	63.70 ± 0.14c	65.55 ± 0.35c
	15	34.48 ± 0.26cd	34.3 ± 0.27c	61.35 ± 0.35d	62.91 ± 0.42d
	20	30.62 ± 0.58d	31.02 ± 0.53d	58.65 ± 0.21e	60.95 ± 0.07e
	0	35.64 ± 0.57bc	37.65 ± 0.56ab	73.85 ± 0.21a	73.40a
	5	37.98 ± 1.12bc	38.57 ± 0.54a	66.80b	68.6 ± 0.14b
Medium	10	38.35 ± 1.34a	38.53 ± 0.09ab	63.65 ± 0.07c	65.85 ± 0.07c
	15	38.21 ± 1.09ab	37.42 ± 0.14b	60.90d	64.05 ± 0.07d
	20	33.46 ± 2.70c	36.07 ± 0.39c	58.22 ± 0.14e	62.80e
	0	35.64 ± 0.57b	37.65 ± 0.56b	73.85 ± 0.21a	73.40a
	5	37.62 ± 0.55a	38.02 ± 0.51a	68.55 ± 0.21b	69.00b
Fine	10	34.48 ± 0.26c	34.30 ± 0.57c	65.35 ± 0.21c	65.75 ± 0.07c
	15	32.66 ± 0.42d	32.45 ± 0.52d	63.35 ± 0.07d	63.60 ± 0.07d
	20	31.8 ± 0.61e	31.74 ± 0.47e	61.15 ± 0.21e	62.10e

*Those with the same letters in the same row indicated that the difference in the same period did not reach the significant level (P > 0.05), while those with different letters indicated that the difference reached the significant level*.

#### Effects of Bran Addition on the Pasting Properties of Flour

RVA starch pasting properties are closely related to the wheat flour processing performance, food texture, and storage aging performance. It affects the quality of steamed bread, noodles, and bread and is an important indicator of starch quality. The effects of bran addition on the pasting properties of flour are shown in Tables 4-1, 4–2. At a constant bran size and with the increase in the addition level of bran, the peak viscosity, trough viscosity, final viscosity, and setback value decreased significantly (*p* < 0.05). Bran could act as a filler in the flour, wherein it reduced the proportion of starch and competed with starch granules for moisture, thus reducing the minimum viscosity values of the bran-containing pastes (28). When the bran concentration was increased, the relative concentration of starch decreased. This effect resulted in a low proportion of expanded starch granules and thus led to a reduction in viscosity. Compared with the control group, the pasting temperature of pastes containing bran were higher. This could be due to the bran absorbed free water, so more energy was needed to destroy the crystal structure of starch granules to make them swell, resulting in an increase in the pasting temperature value (29). As the grain size of the bran increased, the peak viscosity of the flour decreased. The breakdown value of the coarse bran flour was significantly lower than that of the fine and medium bran flours (*p* < 0.05). The size of the bran had no noticeable effect on the minimum and final viscosities of the flour. The setback value is related to the tendency for paste retrogradation. Starch retrogradation occurs in the cooling stage, wherein the amorphous structure of amylose and amylopectin begins to recrystallize through hydrophobic interactions and hydrogen bonds. The competition of the bran for water leads to the redistribution of water in the paste system, resulting in a decrease in starch retrogradation (30). Given that the water retention capacity of fine bran was lower than that of coarse bran, the effect of starch retrogradation inhibition was poor and resulted in a high setback value.

**Table 4-1:** The pasting properties t in CK bran flours.

**Bran granularity**	**Bran addition**	**Peak viscosity**	**Trough viscosity**	**Breakdown**	**Final viscosity**	**Setback**	**Peak time**	**Pasting temperature**
	**(%)**	**(RVU)**	**(RVU)**	**(RVU)**	**(RVU)**	**(RVU)**	**(min)**	**(°C)**
	0	137.21 ± 1.43a	114.67 ± 1.41a	42.5 ± 1.18a	207.92 ± 2.24b	93.25 ± 0.82bc	6.3 ± 0.05a	67.79 ± 0.58c
	5	119.79 ± 0.41c	75.92 ± 1.06d	43.88 ± 0.65a	178.79 ± 2.65d	102.88 ± 1.59b	6.05 ± 0.35a	70.45 ± 0.07b
Fine	10	121.42 ± 3.65a	95.5 ± 3.18bc	41.92 ± 0.47a	230.83 ± 3.54a	135.33 ± 0.35a	6.11 ± 0.12a	75.5 ± 2.12a
	15	120.96 ± 3.59b	85.71 ± 10.31cd	40.25 ± 6.83a	171.58 ± 5.07d	90.88 ± 8.31c	6.08 ± 0.12a	76.3 ± 2.83a
	20	122.08 ± 1.89b	101.54 ± 0.77ab	40.54 ± 2.65a	194.29 ± 0.41c	92.75 ± 1.18bc	6.08 ± 0.26a	78.8 ± 0.42a
	0	137.21 ± 1.43c	114.67 ± 2.03a	42.5 ± 1.18a	207.92 ± 1.58c	93.25 ± 1.52bc	6.3 ± 0.05a	67.79 ± 0.58b
	5	161.00 ± 1.21c	112.5 ± 1.09a	48.50 ± 0.65a	212.92 ± 3.11b	100.42 ± 0.79b	6.27 ± 0.35a	67.45 ± 0.07b
Medium	10	159.83 ± 0.62b	116.5 ± 0.41a	43.50 ± 0.47a	235.83 ± 1.54a	119.5 ± 0.2.33a	6.13 ± 0.07a	72.5 ± 2.12a
	15	134.83 ± 3.08c	96.83 ± 3.541b	40.25 ± 6.83a	187.08 ± 3.21d	90.25 ± 1.83c	6.20 ± 0.12a	76.3 ± 2.83a
	20	150.92 ± 2.40b	94.42 ± 3.187b	44.08 ± 2.65a	182.33 ± 0.91d	87.92 ± 2.01bc	6.19 ± 0.26a	78.8 ± 0.42a
	0	137.21 ± 1.43d	114.67 ± 1.41d	42.5 ± 1.18a	207.92 ± 2.24d	93.25 ± 0.82bc	6.3 ± 0.05a	67.79 ± 0.58a
	5	200.42 ± 1.21c	158.25 ± 1.09b	42.17 ± 0.65a	246.92 ± 3.11b	88.67 ± 0.79b	6.60 ± 0.35a	65.40 ± 0.07a
Coarse	10	229.92 ± 0.62b	186.67 ± 0.41a	43.25 ± 0.47a	306.67 ± 1.54a	120 ± 0.2.33a	6.65 ± 0.07a	64.45 ± 2.12b
	15	192 ± 3.08c	147.17 ± 3.541c	44.83 ± 6.83a	241.17 ± 3.21d	94 ± 1.83c	6.70 ± 0.12a	63.55 ± 2.83b
	20	191.75 ± 2.40b	143.58 ± 3.18c	48.17 ± 2.65a	238.5 ± 0.91c	94.72 ± 2.01bc	6.80 ± 0.26a	61.05 ± 0.42c

*At the same granularity, the same alphabets in right side of the same lift of the item of one stage show no significance (P > 0.05), on the contrary, having significance in same column mean significant among treatments at 5% level*.

**Table 4-2:** The pasting properties t in Zn bran flours.

**Bran granularity**	**Bran addition**	**Peak viscosity**	**Trough viscosity**	**Breakdown**	**Final viscosity**	**Setback**	**Peak time**	**Pasting temperature**
	**(%)**	**(RVU)**	**(RVU)**	**(RVU)**	**(RVU)**	**(RVU)**	**(min)**	**(°C)**
	0	140.96 ± 2.3a	108.46 ± 9.84a	39.67 ± 5.77a	191.96 ± 4.89a	103.50 ± 8.37a	6.30 ± 0.14a	70.83 ± 0.95a
	5	137.96 ± 10.9b	101.29 ± 3.48b	37.5 ± 1.06b	181.5 ± 3.65b	80.21 ± 6.19c	6.47 ± 0.28a	66.98 ± 0.60b
Fine	10	137.08 ± 3.65b	99.50 ± 0.94b	36.58 ± 4.60bc	184.63 ± 5.01b	94.13 ± 5.95b	6.20 ± 0.19a	62.78 ± 0.67c
	15	129.69 ± 6.05c	73.13 ± 1.47d	35.58 ± 1.06c	175.42 ± 0.47c	96.27 ± 1.03b	6.18 ± 0.07a	70.75 ± 0.78a
	20	108.71 ± 2.53d	89.17 ± 6.36c	33.5 ± 0.35d	170.58 ± 5.66c	101.42 ± 0.71a	6.20 ± 0.02a	69.07 ± 0.94a
	0	140.96 ± 2.3b	101.29 ± 3.48b	39.67 ± 5.77a	191.96 ± 4.89a	90.67 ± 8.37c	6.30 ± 0.14a	70.83 ± 0.95a
	5	123.08 ± 10.9c	88.25 ± 9.84a	38.83 ± 1.06b	171.25 ± 5.66b	80.42 ± 6.19d	6.27 ± 0.28a	67.18 ± 0.60b
Medium	10	151.75 ± 3.65a	111.75 ± 0.94b	34 ± 4.60c	166.33 ± 5.01c	114.58 ± 5.95a	6.20 ± 0.19a	61.75 ± 0.67c
	15	119.71 ± 2.53d	80.83 ± 1.47d	38.17 ± 1.06ab	163.42 ± 0.47d	82.58 ± 1.03c	6.07 ± 0.07a	71.05 ± 0.78a
	20	126.67 ± 6.05bc	85.33 ± 6.36c	34.33 ± 0.35c	168.27 ± 3.65c	85.92 ± 0.71bc	6.13 ± 0.02a	69.07 ± 0.94a
	0	140.96 ± 2.3d	101.29 ± 3.48b	39.67 ± 5.77c	191.96 ± 4.89c	90.67 ± 8.37b	6.30 ± 0.14a	70.83 ± 0.95a
	5	192.42 ± 10.9b	135.08 ± 9.84a	40.75 ± 4.60c	247.83 ± 3.65b	112.75 ± 6.19a	6.27 ± 0.28a	65.70 ± 0.60b
Coarse	10	212.67 ± 3.65a	171.92 ± 0.94b	44.42 ± 1.06b	278.92 ± 5.01a	107.00 ± 5.95b	6.80 ± 0.19a	64.45 ± 0.67c
	15	169.00 ± 2.53c	124.58 ± 1.47d	44.83 ± 0.35b	213.58 ± 0.47bc	89.88 ± 1.03d	6.47 ± 0.07a	64.50 ± 0.78c
	20	171.17 ± 6.05bc	126.33 ± 6.36c	57.33 ± 1.06a	215.00 ± 5.66bc	88.67 ± 0.71d	6.47 ± 0.02a	61.05 ± 0.94d

*At the same granularity, the same alphabets in right side of the same lift of the item of one stage show no significance (p > 0.05), on the contrary, having significance in same column mean significant among treatments at 5% level*.

#### Effects of Bran Addition on the Zn Content of Flour

The Zn content of flour with different addition levels and particle sizes of bran are summarized in Table 5. After bran addition, the Zn content of flour increased significantly. The Zn content of bran flour increased significantly (*p* < 0.05) with the increment in the addition level of coarse, medium, and fine bran. Compared with that of the flour without bran, the Zn concentrations of the flour in the Zn fertilizer groups and control groups had enhanced by 5.6–21.7% and 4.35–33.35%, respectively. Obviously, the addition of bran effectively increased the Zn content of flour, and the Zn content of Zn fertilizer flour was significantly higher than that of CK flour (*p* < 0.05). In addition, the different grain sizes of bran had no significant effect on the Zn content of flour.

**Table 5:** The zinc content of the bran flour.

**Bran granularity**	**Bran addition (%)**	**Zinc content (mg/kg)**	
		**CK**	**Zn**
	0	7.50 ± 0.57d	11.85 ± 0.07d
	5	15.05 ± 0.64c	24.45 ± 2.9c
Fine	10	17.00 ± 1.13c	27.95 ± 0.92c
	15	21.85 ± 1.06b	33.65 ± 1.48b
	20	29.05 ± 0.92a	40.85 ± 0.78a
	0	7.50 ± 0.57d	11.85 ± 0.07e
	5	13.10 ± 0.14c	24.20 ± 0.14d
Medium	10	16.10 ± 0.42bc	26.75 ± 0.21c
	15	19.65 ± 0.78b	32.65 ± 1.48b
	20	29.01 ± 2.26a	39.30 ± 0.28a
	0	7.50 ± 0.57e	11.85 ± 0.07e
	5	13.70 ± 0.71d	21.90 ± 0.42d
Coarse	10	16.35 ± 0.07c	26.00 ± 0.42c
	15	29.20 ± 0.42b	32.25 ± 0.21b
	20	28.75 ± 0.07a	37.65 ± 1.06a

*At the same granularity, the same alphabets in right side of the same lift of the item of one stage show no significance (P > 0.05), on the contrary, having significance in same column mean significant among treatments at 5% level*.

### Effect of Bran Addition on Steamed Bread Quality

#### Effects of Bran Addition on the Zn Content of Steamed Bread

The effects of bran addition on the Zn content of steamed bread are shown in Table 6. The trend of Zn content in steamed bread was consistent with that of flour after bran addition. Specifically, both improved significantly with the increase in the addition level of bran (*p* < 0.05). When the amount of bran exceeded 10%, the change in the Zn content of steamed bread in the Zn fertilizer group was no longer obvious. The Zn content of the steamed bread without bran increased by 73% relative to that of the control group. At the 5% bran addition level, the Zn content of the steamed bread in the Zn fertilizer group increased by 55% and that of the control group increased by 29% on average. At the 20% bran addition level, the average Zn content of steamed bread had improved by almost 106% and that of the CK group increased by 131% on average. Therefore, bran addition had an excellent effect on the Zn content of steamed bread. As the bran retains most of the minerals in grains, it seems to favor the use of integral flour for the manufacture of bread and pasta products.

**Table 6:** The zinc content of the bran steamed bread.

**Bran granularity**	**Bran addition (%)**	**Zinc content (mg/kg)**	
		**CK**	**Zn**
	0	16.05 ± 0.78e	25.75 ± 4.59c
	5	20.20 ± 0.14d	40.04 ± 0.42b
Fine	10	27.45 ± 0.21c	47.19 ± 0.7ab
	15	34.00 ± 1.13b	50.35 ± 0.21a
	20	37.91 ± 0.71a	53.90 ± 0a
	0	16.05 ± 0.78e	25.75 ± 4.6c
	5	19.90 ± 0.28d	40.15 ± 1.41b
Medium	10	26.71 ± 1.84c	46.25 ± 3.18ab
	15	31.50 ± 0.28b	49.9 ± 1.27a
	20	36.00 ± 0.71a	53.05 ± 0.78a
	0	16.05 ± 0.78d	25.75 ± 4.6c
	5	21.75 ± 0.25c	39.25 ± 0.35b
Coarse	10	26.47 ± 0.32bc	46.72 ± 0.64ab
	15	32.20 ± 2.83b	49.2 ± 0.14ab
	20	37.25 ± 1.21a	52.75 ± 4.59a

*At the same granularity, the same alphabets in right side of the same lift of the item of one stage show no significance (P > 0.05), on the contrary, having significance in same column mean significant among treatments at 5% level*.

#### Effects of Bran Addition on the Specific Volume of Steamed Bread

**Figure 3:**
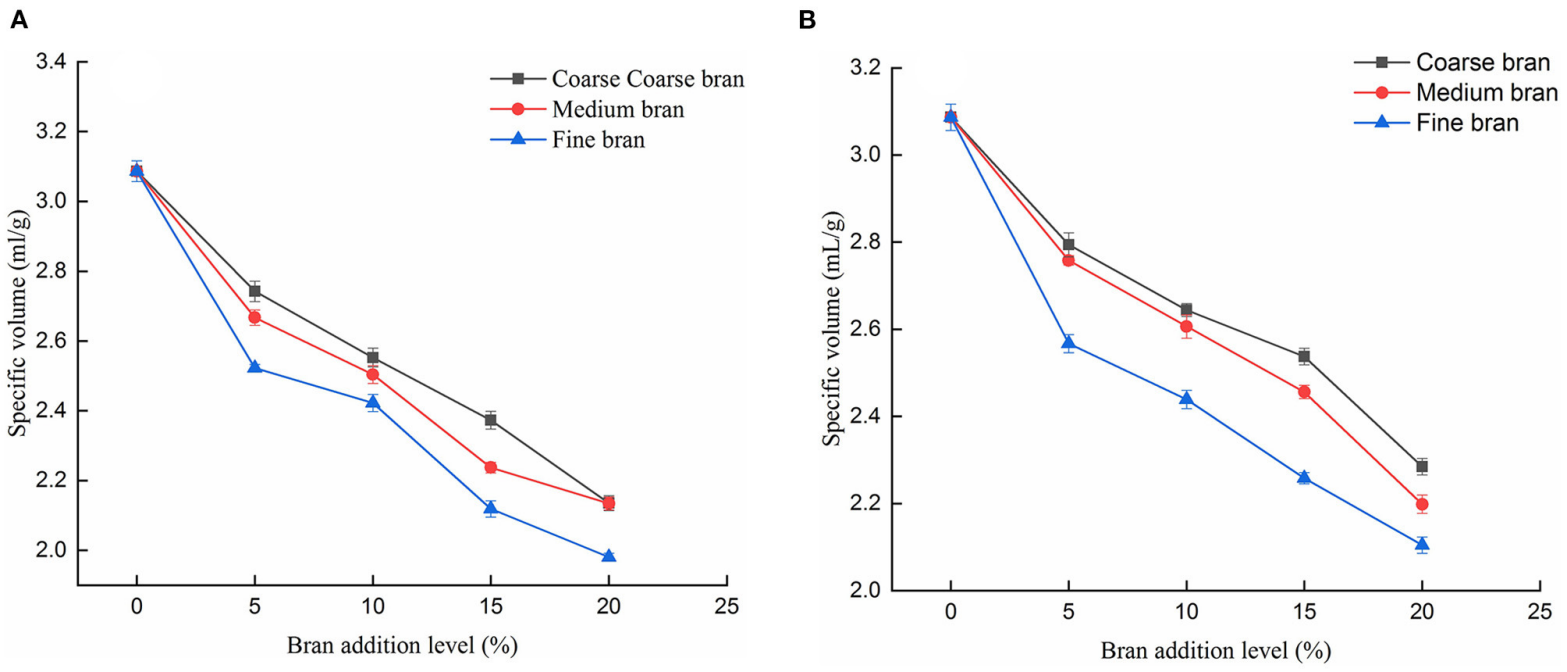
Effect of bran addition on the specific volume of steamed bread. **(A)** control group; **(B)** Zn fertilizer group.

As can be seen from Figure 3, the specific volume of the steamed bread in the control group (Figure 3A) and the Zn fertilizer group (Figure 3B) decreased with the increase in bran addition. Meanwhile, with the reduction of bran particle size, the specific volume also decreased. Similar result was reported that steamed breads containing smaller size bran had lower specific volume (31). Small bran particles could inhibit the formation of gluten network structure and reduce the gas fixed in the air chamber, and affect the height and volume of the steamed bread (27). The filling of the three-dimensional space structure of the starch–protein matrix increased density. This effect resulted in a reduction in the volume and specific volume of the steamed bread. This trend confirmed the finding that higher bran addition (>7%) led to decreased expansion of dough during kneading (32). Therefore, the optimal bran addition level was 5%.

#### Effects of Bran Addition on the Texture of Steamed Bread

The textural parameters of steamed bread with different bran additions as obtained through TPA are shown in Table 7-1, 7-2. Compared with those of steamed bread without bran, the hardness, gumminess, and chewiness of steamed bread with bran increased with the increase in bran addition, whereas springiness and cohesiveness declined likely because the steric hindrance of bran caused gluten protein dilution (4). Moreover, brain contained rigid dietary fibers and would thus compete with flour in water absorption. The water absorption of bran fiber and the gelatinization of starch during the steaming process increase the viscosity, which may be related to the gumminess and chewiness. In addition, with the decrement in the particle size of bran, the hardness, gumminess, and chewiness of steamed bread increased significantly, whereas springiness and chewiness decreased significantly (*p* < 0.05). Hardness is usually closely related to product acceptability (33). For steamed buns, the soft texture is an ideal quality characteristic. There was a study found that smaller bran particle size resulted in significantly lower hardness in flat bread (34). On the contrary, Li et al. (31) indicated that bread made by mixing hard white flour with fine bran has higher hardness than bread prepared with coarse and medium bran, which was similar to the results of our experiment. This probably because of steamed bread with lower specific volume had denser structure and more compact gas cells and thus increased the steamed bun hardness. The best texture of steamed bread was obtained at the bran addition level of 5%. By contrast, when the amount of bran exceeded 10%, the textural parameters showed significant reductions, and the internal structure was destroyed. In general, the steamed wheat bread added with 5% medium bran was selected as the best product. The results of this study showed that the control group and the foliar Zn group had the same trends.

**Table 7-1:** Texture analysis of CK bran steamed bread.

**Granularity**	**Bran addition/%**	**Hardness/g**	**Springiness/%**	**Cohesiveness**	**Gumminess**	**Chewiness**
	0	2903.01 ± 57.28e	0.98a	0.85a	2519.32 ± 110.46e	2298.91 ± 120.09e
	5	3641.23 ± 213.01d	0.97b	0.85a	2835.29 ± 61.11d	2667.12 ± 149.28d
Coarse	10	5219.12 ± 282.84c	0.96c	0.84ab	4215.29 ± 205.03c	3871.06 ± 6.98c
	15	6463.96 ± 70.71b	0.94d	0.83ab	5181.51 ± 513.02b	5075.59 ± 180.52b
	20	7410 ± 217.54a	0.93e	0.81b	6228.03 ± 342.17a	5749.17 ± 107.89a
	0	2903.01 ± 57.28e	0.98a	0.85a	2519.32 ± 110.46e	2298.91 ± 120.09e
	5	4432.88 ± 114.02b	0.96ab	0.84b	3598.16 ± 358.75c	3452.9 ± 310.71c
Medium	10	5149.14 ± 104.89c	0.94bc	0.83c	4176.20 ± 174.17c	4039.01 ± 316.75c
	15	7222.15 ± 24.77d	0.94c	0.81d	5568.32 ± 296.74b	5286.76 ± 158.45b
	20	8821.58 ± 25.69e	0.92c	0.8e	6793.77 ± 291.42a	6366.42 ± 327.36a
	0	2903.01 ± 57.28e	0.98a	0.85a	2519.32 ± 110.46e	2298.91 ± 120.09e
	5	4649.69 ± 7.4d	0.95b	0.83b	3836.78 ± 21.3d	3650.63 ± 31.07d
Fine	10	6146.43 ± 169.51c	0.93bc	0.80c	4911.46 ± 131.11c	4492.99 ± 112.14c
	15	8245.61 ± 137.45b	0.92c	0.77d	6179.10 ± 123.33b	6650.93 ± 114.65b
	20	9602.97 ± 315.87a	0.89d	0.75e	7105.42 ± 149.32a	7053.01 ± 175.52a

*At the same granularity, the same alphabets in right side of the same lift of the item of one stage show no significance (P > 0.05), on the contrary, having significance in same column mean significant among treatments at 5% level*.

**Table 7-2:** Texture analysis of Zn bran steamed bread.

**Granularity**	**Bran addition/%**	**Hardness/g**	**Springiness/%**	**Cohesiveness**	**Gumminess**	**Chewiness**
	0	2674.66 ± 56.74e	0.97a	0.85a	2512.56 ± 30.25e	2257.04 ± 60.88e
	5	3355.33 ± 5.94d	0.96a	0.84ab	3036.01 ± 11.68d	2871.42 ± 20.34d
Coarse	10	4811.09 ± 23.21c	0.95ab	0.83b	4424.42 ± 57.78c	3972.28 ± 6.36c
	15	6420.49 ± 132.83b	0.93bc	0.81c	5390.94 ± 14.75b	4760.12 ± 65.63b
	20	7710.97 ± 251.89a	0.91c	0.77d	5706.35 ± 78.72a	5323.7 ± 38.82a
	0	2674.66 ± 56.74e	0.97a	0.85a	2512.56 ± 30.25e	2257.04 ± 60.88e
	5	4008.29 ± 140.86d	0.96b	0.85ab	3324.81 ± 11.73c	3144.03 ± 58.25d
Medium	10	5260.99 ± 61.20c	0.94c	0.83b	4159.03 ± 49.90b	4346.87 ± 105.41c
	15	7722.87 ± 141.25b	0.93d	0.81c	5909.49 ± 64.42a	5329.59 ± 82.28b
	20	8205.33 ± 91.96a	0.92e	0.80d	6240.47 ± 36.54a	5846.52 ± 128.10a
	0	2674.66 ± 56.74e	0.97a	0.85a	2512.56 ± 30.25e	2257.04 ± 60.88e
	5	4762.42 ± 7.40d	0.95a	0.81b	3798.44 ± 51.20d	3562.53 ± 49.28d
Fine	10	6346.43 ± 169.51c	0.93b	0.79c	5010.65 ± 122.11c	4502.99 ± 107.14c
	15	8310.21 ± 47.32b	0.91c	0.77d	6157.32 ± 89.35b	6579.42 ± 111.75b
	20	9588.75 ± 125.24a	0.88d	0.76e	7028.33 ± 152.04a	6971.21 ± 169.20a

*At the same granularity, the same alphabets in right side of the same lift of the item of one stage show no significance (P > 0.05), on the contrary, having significance in same column mean significant among treatments at 5% level*.

#### Effects of Bran Addition on the Sensory Evaluation of Steamed Bread

The sensory scores of the steamed breads indicated that specific volume, surface color, appearance shape, internal structure, spring, tenacity, and odor were affected by the different addition levels and particle sizes of bran The physicochemical properties of wheat bran are quite different from those of refined flour and lead to inferior product quality (with regard to appearance, flavor, and sensory acceptance) of bran dough-based products (35). The sensory scores of the steamed breads with different bran additions are shown in Table 8-1, 8-2. The addition of bran could affect the fermentation and air-holding capacity of the dough. Thus, the volume and specific volume of steamed bread changed with bran addition. With the addition of fine bran, the taste of steamed bread worsened, and the smell of steamed bread became unpleasant. Moreover, considering the close fusion of the small-grained bran with the flour, the inner structure of the dough became dense and the unfolding of the gluten network structure was hindered. These effects resulted in the formation of excessively small pores or even prevented the formation of pores. With the addition of coarse bran, the surface of the steamed breads became obviously granular and thus caused distinct discomfort during chewing and swallowing. When the added amount of coarse bran exceeded 5%, the steamed bread exhibited a rough texture and loose internal structure. The total scores of the samples indicated that the steamed bread containing medium-sized bran at the 5% addition level had the highest sensory score and resulted in comparable flavor, and texture relative to control. Numerous negative effects of bran addition on dough and product properties have been reported such as: darker color, dough stickiness increase, specific volume reduction, coarser texture and so on (36). The main problem is the consumer's acceptability of Zn-enriched bran steamed bread. On the basis of the sensory scores, flour physicochemical properties, Zn content, and acceptability of the steamed bread samples, the steamed bread with 5% medium bran was identified as the best option under the conditions of this experiment. This result provides scientific knowledge for guiding the production of steamed bread with bran fortified through Zn fertilization.

**Table 8-1:** The sensory scores of CK bran steamed bread.

**Sample**	**Specific volume**	**Surface color**	**Surface structure**	**Appearance shape**	**Internal structure**	**Spring**	**Tenacity**	**Viscosity**	**Odor**	**Total**
	**(20)**	**(10)**	**(10)**	**(10)**	**(15)**	**(10)**	**(10)**	**(10)**	**(10)**	**score**
CK	20	9.2	9.0	9.0	13.5	8.5	8.5	8.5	4.7	90.9
CK-C-5	17	8.5	8.0	8.3	10.4	8.0	7.5	8.1	4.8	80.6
CK-C-10	16	7.4	6.5	7.2	9.0	7.5	8.0	7.2	4.5	73.3
CK-C-15	15.5	6.8	7.0	6.0	9.2	7.2	7.0	6.0	4.5	69.2
CK-C-20	14.3	6.0	6.0	5.0	8.7	6.6	6.0	5.8	4.3	62.7
CK-M-5	17	8.5	9.0	8.2	10.6	8.0	8.0	8.5	4.8	82.6
CK-M-10	16	7.5	8.0	8.0	9.9	7.2	8.0	7.2	4.7	76.5
CK-M-15	15.7	6.2	7.3	7.2	8.5	7.0	6.5	6.3	4.6	69.3
CK-M-20	13.2	5.2	7.0	6.5	7.8	6.2	5.0	5.5	4.5	60.9
CK-F-5	16.7	9.0	8.0	8.8	11.7	7.6	6.0	7.5	4.3	79.6
CK-F-10	15	7.7	8.0	8.1	11.0	7.4	5.7	7.4	3.2	73.5
CK-F-15	13.5	6.3	7.0	8.0	7.2	6.2	5.0	6.2	2.7	62.1
CK-F-20	13	5.6	6.0	4.0	6.3	5.0	4.0	4.3	2.0	50.2

*CK-C-5, CK flour with 5% coarse bran addition level; CK-M-5, CK flour with 5% medium bran addition level; CK-F-5, CK flour with 5% fine bran addition level and so on*.

**Table 8-2:** The sensory scores of Zn bran steamed bread.

**Sample**	**Specific volume**	**Surface color**	**Surface structure**	**Appearance shape**	**Internal structure**	**Spring**	**Tenacity**	**Viscosity**	**Odor**	**Total score**
	**(20)**	**(10)**	**(10)**	**(10)**	**(15)**	**(10)**	**(10)**	**(10)**	**(10)**	
Zn	20	9.1	10.0	9.1	13.5	8.8	8.5	8.5	4.5	92
Zn-C-5	18	8.7	8.0	8.3	10.5	8.5	7.5	8.0	4.8	82.3
Zn-C-10	16.2	8.5	6.5	7.4	10.5	7.6	8.1	7.0	4.6	74.4
Zn-C-15	15.5	7.4	7.0	6.2	10.3	7.2	7.0	6.2	4.2	71
Zn-C-20	14.3	6.3	6.0	5.1	9.4	8.7	6.5	6.0	4.0	66.3
Zn-M-5	17.8	8.8	9.0	8.8	11.0	8.2	8.0	8.0	4.8	84.4
Zn-M-10	16	7.8	8.0	8.5	10.4	7.0	8.2	7.0	4.8	77.7
Zn-M-15	15.8	6.4	7.5	7.2	9.8	7.2	6.5	6.3	4.6	71.3
Zn-M-20	14.7	6.0	7.0	7.0	9.0	6.2	5.0	5.0	4.5	64.4
Zn-F-5	16.4	9.0	8.0	8.8	11.8	8.8	6.0	7.5	4.2	80.5
Zn-F-10	15.5	7.7	8.0	8.0	10.4	7.5	5.7	7.2	3.5	73.5
Zn-F-15	14.2	5.6	7.0	7.8	7.5	6.0	5.0	6.4	3.0	62.5
Zn-F-20	13	5.0	6.0	4.5	6.0	5.5	4.5	4.0	2.0	50.5

*Zn-C-5, Zn flour with 5% coarse bran addition level; Zn-M-5, Zn flour with 5% medium bran addition level, Zn-F-5, Zn flour with 5% fine bran addition level and so on*.

## Conclusion

As a result of the foliar application of ZnSO4, the Zn content of wheat grains and flour increased by 47.4 and 58.7%, respectively, which met the target levels for Zn biofortification. The addition level and particle size of wheat bran affected the mixing characteristics and pasting properties of flour and thus weakened mixing tolerance. The addition of wheat bran significantly decreased peak viscosity, trough viscosity, final viscosity, and setback value (*p* < 0.05). However, particle size had no significant effect on pasting properties. The whiteness and L^*^ of the mixed flour gradually decreased with the increment in the particle size and addition amount of bran. The hardness, gumminess, and chewiness of steamed bread showed an upward trend with the increase in bran addition level, whereas springiness and cohesiveness declined. The sensory total scores of steamed bread in the control and Zn fertilizer groups were optimal at the 5% bran addition level. By adding 5% medium bran to wheat flour, the Zn content of the steamed bread in the Zn fertilizer group reached 40.2 mg/kg, whereas that of the steamed bread in the control group was only 25.8 mg/kg. The Zn content of the steamed bran bread was 55.8% higher than that of the control group. This increment indicated that the Zn content of this staple food was enhanced. In conclusion, the results of this study could be useful in the application of wheat bran as an ingredient for enhancing the content Zn of steamed bread. On one hand, we offer a sustainable and low-cost way to provide essential micronutrients (Zn) to people in both developing and developed countries. On the other hand, wheat bran as the main by-product of wheat milling usually has low value but in this way it can be a good source of Zn. The influence of bran on steamed bread structure, nutritional ingredients, and qualities need to be further researched. Meanwhile, further research is needed to minimize the detrimental influence of bran on the quality and sensory acceptance of bran addition products, such as applying physical mode (extrusion, autoclaving and autoclaving) to modify the functional properties of bran and enhance the consumption of bran dough-based products.

## Data Availability Statement

The original contributions presented in the study are included in the article/supplementary material, further inquiries can be directed to the corresponding author/s.

## Author Contributions

HW and XZ contributed to the design, implementation, and data analysis of the study. HW conducted the experiments, analyzed the data, and wrote the first draft of the manuscript. XZ supervised the findings of this work, verified data analysis, and contributed to the interpretation of results and discussion. All authors contributed to the final manuscript and approved the final version.

## Funding

This work was supported by Agricultural Variety Improvement Project of Shandong Province (2019LZGC001).

## Conflict of Interest

The authors declare that the research was conducted in the absence of any commercial or financial relationships that could be construed as a potential conflict of interest.

## Publisher's Note

All claims expressed in this article are solely those of the authors and do not necessarily represent those of their affiliated organizations, or those of the publisher, the editors and the reviewers. Any product that may be evaluated in this article, or claim that may be made by its manufacturer, is not guaranteed or endorsed by the publisher.

## Abbreviations

ICP-MS: Inductively coupled plasma mass spectrometry
1M: Core powder 1
2M: Core powder 2
3M: Core powder 3
1B: Hide powder 1
2B: Hide powder 2
3B: Hide powder 3.
