# Supplementary material for: Effect of Zn-Rich Wheat Bran With Different Particle Sizes on the Quality of Steamed Bread
Source: Front Nutr. 2021 Dec 10;8:761708. doi: 10.3389/fnut.2021.761708 (PMC8702855; doi:10.3389/fnut.2021.761708)

The following is the Supplementary data to this article:

The side views and cross-sectional views of Zn-rich wheat bran steamed bread


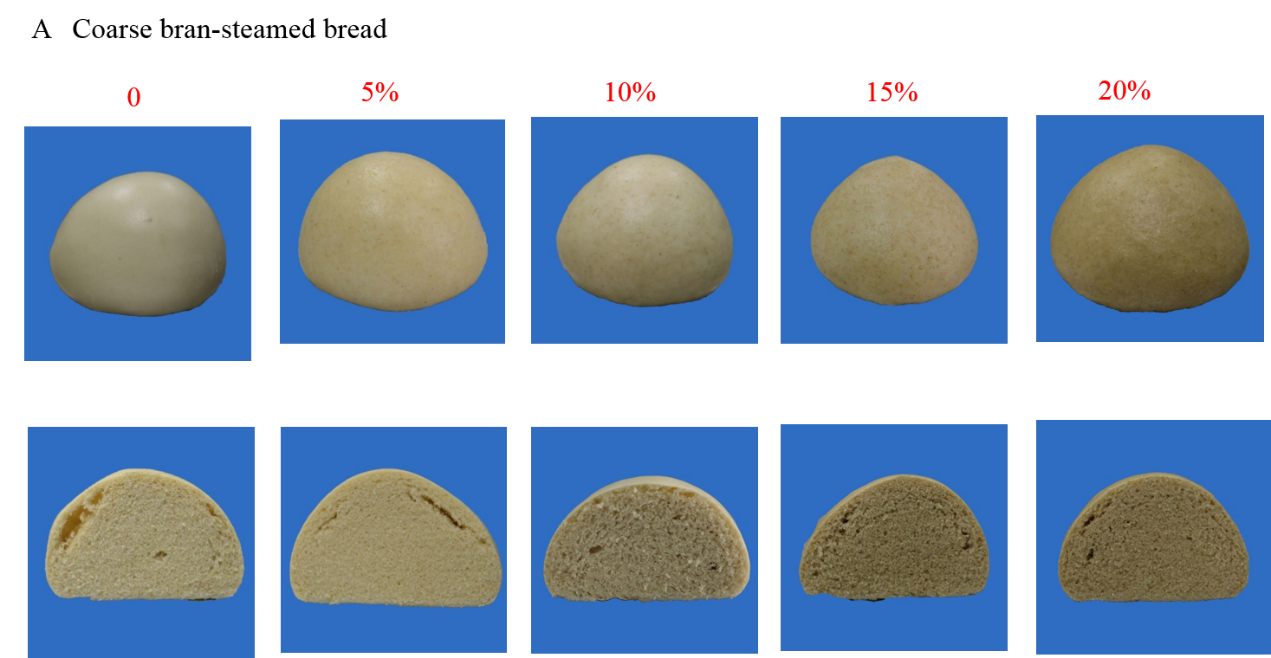


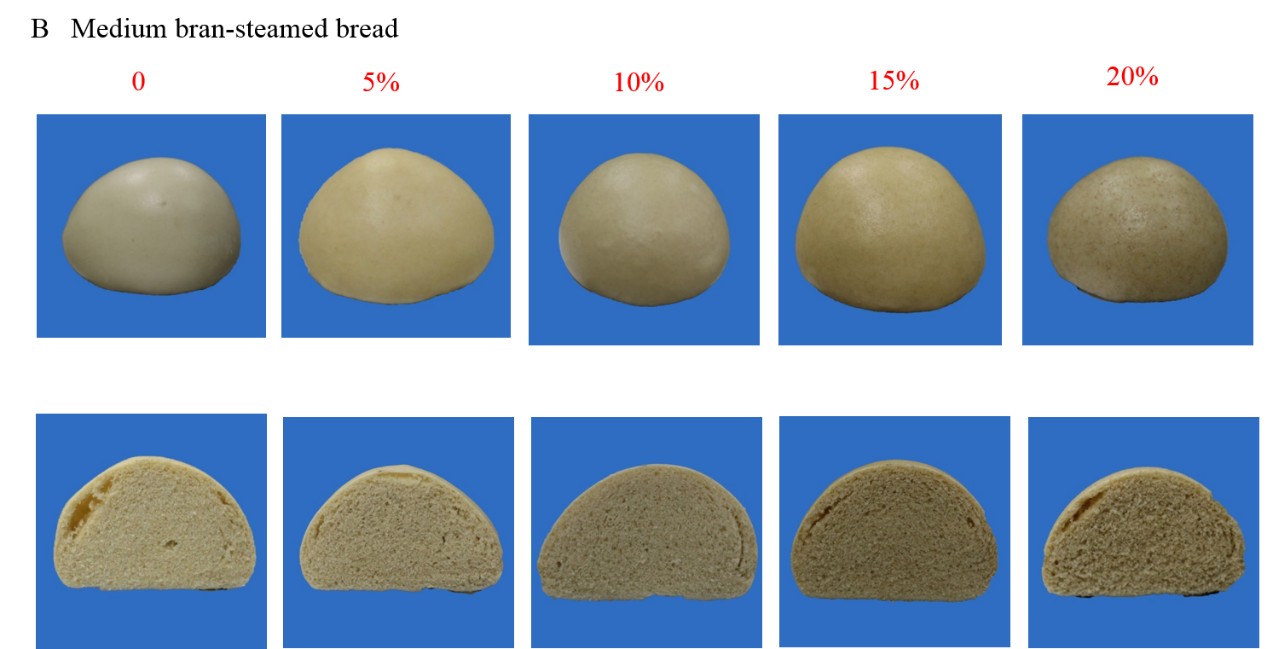


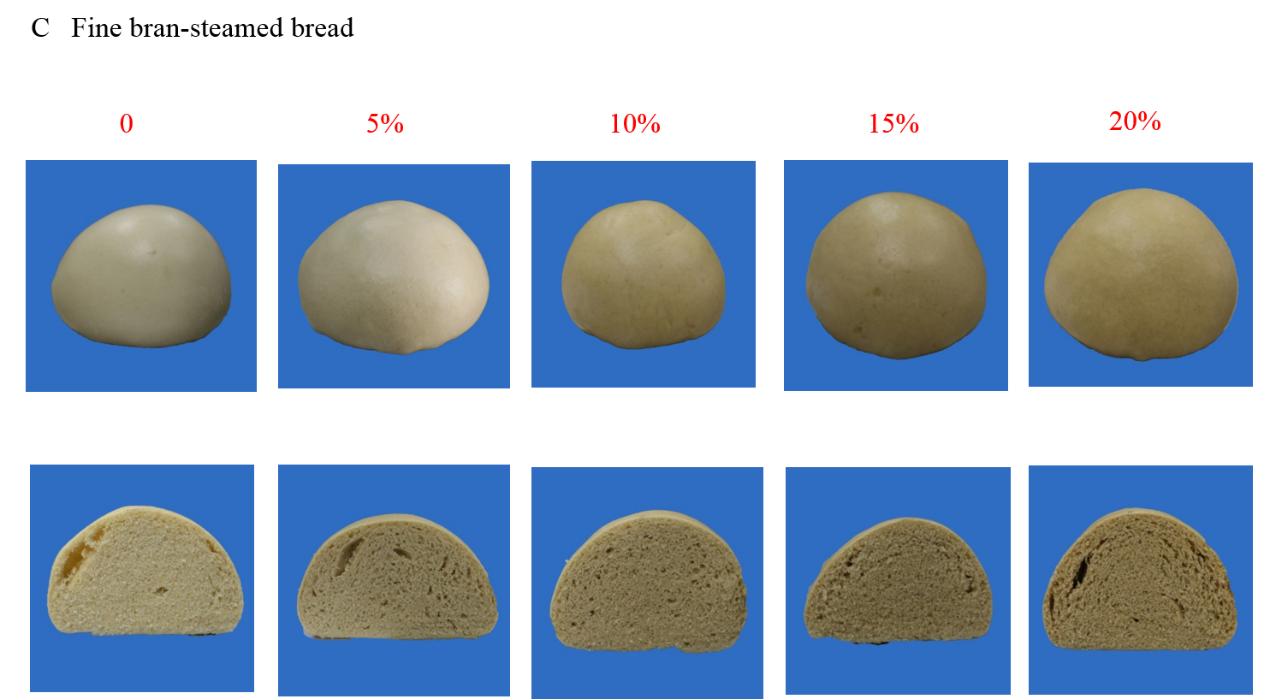

Supplement: Supplementary file 1 [file Data_Sheet_1.docx]
